# Simultaneous Determination of Ascorbic Acid, Uric Acid and Tryptophan by Novel Carbon Nanotube Paste Electrode

**Published:** 2018

**Authors:** Mohammad Mazloum-Ardakani, Mahboobe Abolhasani-Soorki, Alireza Khoshroo, Fariba Sabaghian, Bibi-Fatemeh Mirjalili

**Affiliations:** *Department of Chemistry, Faculty of Science, Yazd University, Yazd, Iran.*

**Keywords:** Sensor, Ascorbic acid, Uric Acid, Tryptophan, Carbon nanotube

## Abstract

In the present paper, electrochemical methods were used to investigate the behavior of ascorbic acid at a carbon paste electrode modified with 2,2′-((1E)-(1,2 phenylenebis(azanylylidene)) bis(methanylylidene))bis(benzene-1,4-diol) (PBD) and oxidized multiwall carbon nanotubes. The modified carbon paste electrode showed high electrocatalytic activity toward ascorbic acid; the current was enhanced significantly relative to the situation prevailing when an unmodified carbon paste electrode was used. Cyclic voltammetry was used to investigate the redox properties of this modified electrode at various solution pH values and at various scan rates. Using differential pulse voltammetry, the calibration curves for AA were obtained over the range of 1.0–80.0 and 80–4000.0 μM, respectively. The detection limit was 0.3 μM. The present method provides a simple method for selective detection of ascorbic acid. DPV also was used for simultaneous determination of AA, uric acid, and tryptophan at the modified electrode. Finally, the proposed electrochemical sensor was used for determinations of these substances in in biological systems and pharmaceutical samples.

## Introduction

**Scheme 1:**
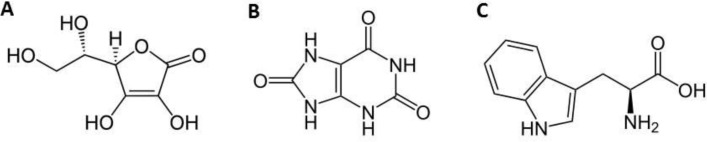
Structure of (A) Ascorbic Acid, (B) Uric Acid and (C) Tryptophan.

Ascorbic acid (AA, Scheme 1A) is consumed worldwide on a large scale as an antioxidant agent in food and beverages and in medicines. It has been used for long at the prevention and treatment of common cold and mental illness (1). Wilson and Guillan (2) have reported abnormalities related to the AA levels in schizophrenic patients. AA is also an important agent in several enzymatic reactions and in cell defense against oxidative stress (3). Oxidative stress is associated to an excessive amount of free radicals, very unstable molecules arising physiologically during cellular aerobic metabolism (2–3% of oxygen consumed by a cell is converted into free radicals) (4) that may lead to disruption of a living cell or to molecular and cellular DNA damage (5). Free radicals have been indicated as probable pathogenesis determinants of many degenerative and chronic diseases that develop with age, such as cancer, cardiovascular disease, cataract, and immunity system dysfunctions. For its potential role in preventing such diseases and food spoilage, researchers and nutritionists have devoted great attention to AA along several decades. 

Among the methods available for the measurement of ascorbic acid we can highlight and high performance liquid chromatography (HPLC) (6), gas chromatography (GC) (7), electrophoresis (8), flow injection spectrophotometry (9), sequential injection analysis (10), calorimetry (11), flame atomic absorption spectrometry (12), kinetic spectrophotometric (13), enzymatic spectrophotometry (14), polarography (15) and titration (16), *etc.* Nevertheless some of the above techniques suffer from diverse disadvantages with regard to the expensive equipment, insufficient selectivity, complicated derivatization for sensitive detection, and time-consuming process of sample clear-up to prevent deteriorating chromatographic columns (17). In this sense, the development of sensors for AA determination is of considerable interest because its use involves several advantages such as rapid response, high specificity, low cost, and no need of sample preparation. Carbon-based electrodes, mainly modified carbon paste electrode (CPE), are the best suited for the determination of compounds present in biological fluids (18-21). 

One of the most important effects of any mediator is a reduction of the overpotential required for electrochemical reaction, which enhances the sensitivity (current) and selectivity of the method (22-25). Carbon paste electrodes constitute a convenient conductive matrix for the preparation of chemically modified electrodes by simple mixing of graphite/binder paste and modifier (26, 27). These kinds of electrode are inexpensive and possess many advantages such as low background current, wide range of potential, easy fabrication, and rapid renewal (28). Carbon nanotubes (CNTs) have attracted much attention during the past decade (29), due to their unique mechanical, chemical, and electrical properties. CNTs with diameters in the range of 5–40 nm and up to several microns in length can now be produced in macro quantities. According to their atomic structure, CNTs behave electrically as a metal or as a semiconductor (30). They have many significant properties such as finite small size, high specific surface area, high porosity and unique physical, chemical and electrical properties and can be used as attractive novel materials in electrochemical fields (31-33). The subtle electronic properties suggest that CNTs should have the ability to mediate electron transfer reactions with electroactive species in solution when used as an electrode. The reactivity of CNTs in solution has been demonstrated, resulting in specific reactive (oxidative) sites on the CNTs surfaces. Thus, an important application of CNTs is that they can be used as the electrode material in CNT paste electrodes or CNT modified glassy carbon electrodes to investigate the electrochemical properties of biomolecules (34-36).

In this paper, we described the preparation and suitability of a modified carbon nanotube pasteelectrode (PBDCNPE) as a new electrocatalyst for the electrocatalysis and determination of AA in an aqueous buffer solution. In addition, we have evaluated the analytical performance of the modified electrode for quantification of AA in the presence of UA and Trp (Scheme 1). Finally, in order to demonstrate the catalytic potential of this modified electrode for electrooxidation of AA in real samples, we have examined this method for the voltammetric determination of AA in the in biological samples.

## Experimental

*Apparatus and reagents*

The electrochemical measurements were performed with a potentiostat/galvanostat (SAMA 500, electroanalyzer, system, Iran). A three-electrode cell was used at room temperature. A saturated calomel electrode, a platinum wire and PBDCNPE were used as reference, auxiliary, and working electrodes, respectively. pH measurements were carried out with a Metrohm model 691 pH/mV meter. All solutions were prepared with doubly distilled water. AA, UA, Trp and other reagents were analytical grade (Sigma-Aldrich). Phosphate buffer solutions (0.1 M) were prepared from 0.1 M H_3_PO_4_–NaH_2_PO_4_, and the pH was adjusted with 0.1 M H_3_PO_4_ or NaOH. Graphite paste was prepared from two main components of graphite powder (Merck) and paraffin oil (DC 350, Merck, density = 0.88 g cm^−3^).

*Synthesis of 2,2’-[1,2–phenylenediyl-bis(nitrilomethylidene)]-bis(4-hydroxyphenol)*

To a mixture of 2,5-dihydroxybenzaldehyde (0.35 g, 2.5 mmol) in MeOH was added 1,2-phenylene diamine (0.15 g, 1.4 mmol) and stirring for 30 min. The progress of the reaction was monitored by TLC. After the reaction completion, a red solid product was filtered off and washed with cold MeOH and the pure desired Schiff base was obtained in 96% yield. The Schiff base product was identified by physical and spectroscopic data as following; 2,2’-[1,2–phenylenediyl-bis(nitrilomethylidene)]-bis(4-hydroxyphenol): Red solid; Yield: 96%; M.p: 270–272 °C. Anal. Calcd.: C, 68.9; H, 4.6; N, 8.04. Found: C, 68.7; H, 4.9; N, 7.7. IR (KBr)/υ(cm^-1^): 3250–3500 (s, br, 2OH), 1619 (s, C=N), 1572, 1488 (Ar), 1289 (s, C-O). ^1^H NMR (400 MHz/DMSO-d6)/δ ppm: 12.13 (br, 2OH, Intramolecular hydrogen bonding), 9.10 (br, 2OH), 8.79 (s, 2CH Imine), 7.40 (dd, 2H, Ar, J_1_ = 8.2 Hz, J_2_ = 2.3 Hz), 7.37 (dd, 2H, Ar, J_1_ = 8.3 Hz, J_2_ = 2.3 Hz), 7.02 (d, 2H, Ar, J = 2.8 Hz), 6.86 (dd, 2H, Ar, J_1_ = 8.1 Hz, J_2_ = 2.7 Hz), 6.78 (d, 2H, Ar, J = 8.8 Hz). ^13^C NMR(100 MHz/DMSO-d6)/δ ppm: 164.28, 153.73, 150.04, 142.98, 128.03, 121.77, 120.28, 119.88, 117.38, 117.27. MS: m/z = 348 (M‏‏^+^, 3), 212 (10), 129 (14), 92 (78), 93 (14), 80 (42), 77 (47), 65 (100). UV/λ_max_: 360 (s), 260 (w). UV/λ_max_: 370 (s), 260 (w). 

*Preparation of oxidized CNTs*

Since the oxygen functionalities on the surface of CNTs improve their electrochemical properties, oxidized CNTs were generated by treating CNTs with a mixture of concentrated H_2_SO_4_ and HNO_3_ (molar ratio 3:1) following the method reported in the literature (37). In a typical experiment, 75.0 mL of concentrated H_2_SO_4_ (97%) and 25.0 mL of concentrated HNO_3_ (65%) were mixed and added to 1.0 g of MWCNTs in a round–bottomed flask and heated under constant agitation at 50.0 ºC for 8.0 h. It was allowed to cool down to room temperature after which an equal quantity of deionized water was added. It was filtered and the residue was washed several times with deionized water until neutral pH was attained. The residue was then filtered and freeze–dried.

*Preparation of the electrode*

The PBDCNPE were prepared by mixing 0.94 g of graphite powder, 0.03 g of PBD, 0.03 g of CNT and 0.7 mL of paraffin oil with a mortar and pestle until a uniformly wetted paste was obtained. These amounts of materials were obtained by optimization. The paste was then packed into the end of a glass tube (ca. 3.5 mm i.d. and 10 cm long). A copper wire inserted into the carbon paste provided an electrical contact. When necessary, the surface of the carbon paste was polished on a smooth paper to obtain a shiny appearance. For comparison, PBD modified CPE electrode without CNTs (PBDCPE), CNT paste electrode without PBD (CNPE), and unmodified CPE in the absence of both PBD and CNT were also prepared in the same way.

## Results and Discussion

*Characterization of PBDCNPE*

**Figure 1:**
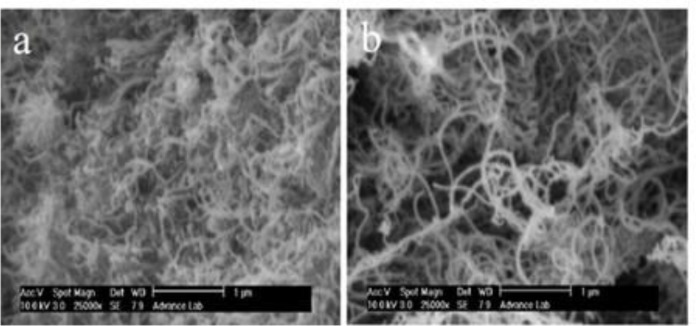
(a) SEM images of MWCNTs before oxidation and, (b) after oxidation.

Scanning electron microscopy was used to detect possible morphological changes on MWCNTs after the treatment. SEM images of MWCNTs and OCNT are shown in Figure 1. The raw MWCNT being strongly entangled it is practically impossible to align them (Figure 1a). As the oxidation proceeds the MWCNTs are gradually freed from the entanglements favoring their alignment. Furthermore the MWCNTs with small diameters are lost (Figure 1b).

**Figure 2:**
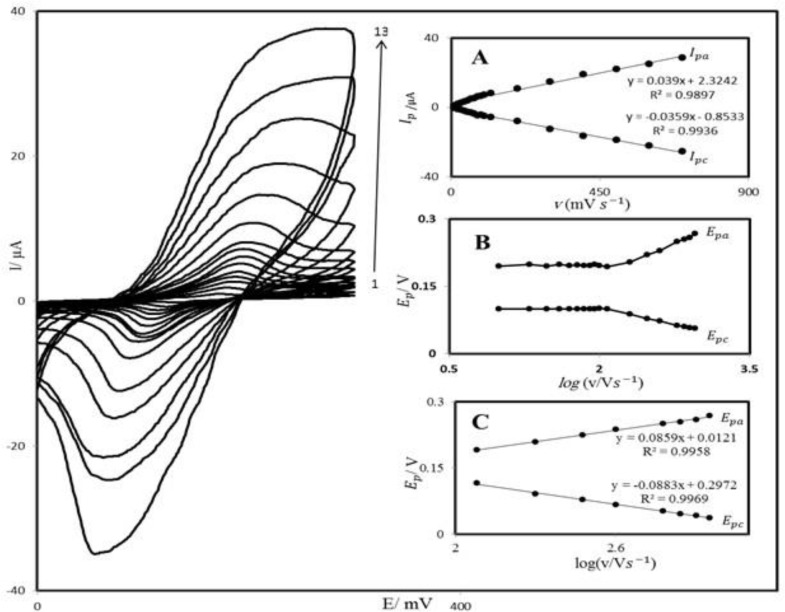
Cyclic voltammograms of PBDCNPE in 0.1 M phosphate buffer (pH 7.0), at various scan rates: the numbers1–13 correspond to 10, 20, 50, 70, 80, 100, 120, 200, 300, 400, 600, 800 and 1200 mV s^−1^ scan rates, respectively. Insets: (A) Variations of I_p_ versus scan rates (B) Variation of E_p_ versus the logarithm of the scan rate. (C) Magnification of the same plot for high scan rates.

PBD compound is insoluble in aqueous media; therefore, we prepared PBDCNPE and studied its electrochemical properties in a buffered aqueous solution (pH 7.0) using cyclic voltammetry. The cyclic voltammograms for the modified electrode at different scan rates in 0.1 M phosphate buffer with pH 7.0 are shown in Figure 2. A pair of reversible peaks are observed at E_pa_ = 0.20 V and E_pc_ = 0.10 V *vs.* SCE and ΔE_p_ = (E_pa _− E_pc_) was 0.10 V. The electrode process was quasi-reversible, with ΔEp, greater than the expected for a reversible system. Inset A of Figure 2 illustrates that the anodic and cathodic peak currents (I_p_) were linearly dependent on υ at scan rates 10–1200 mV s^−1^. A linear correlation was obtained between peak currents, and the scan rate indicates that the nature of redox process was controlled in a diffusion-independent manner (Figure 2A).

An approximate estimate of the surface coverage (Γ) of the modified carbon paste electrode, given in mol cm^−2^, was made by adopting the method used by Sharp *et al.* (38). According to this method, the peak current is related to the surface concentration of electroactive species, by the following Equation:

I_p_ = n^2^F^2^AΓ*v*/4RT                    (1) 

Where n represents the number of electrons involved in reaction, A (cm^2^) is the surface area of the PBDCNPE, Γ (mol cm^−2^) is the surface coverage and other symbols have their usual meanings. From the slope of anodic peak currents versus scan rate (Figure 2B) the calculated surface concentration of PBD is Γ = 8.218 × 10^-8^ mol cm^−2^ for n = 2. Laviron (39) derived general expressions for the linear potential sweep voltammetric response of surface-confined electroactive species: 

log k_s_ = α log (1 − α) + (1 − α) log α − log (RT/n_α_Fυ) – α (1 − α) n_α_F∆E_p_/2.3RT                      (2)

A plot of E_p_ as a function of log υ yields one straight line with a slope equal to 2.3RT/(1 – α)nF for the anodic peak (Figure 2C). Using such a plot and Equation 2, the values of α and k_s_ were determined to be 0.31 and 1.17 s^-1^, respectively.

*Effect of pH on peak potential*

**Figure 3:**
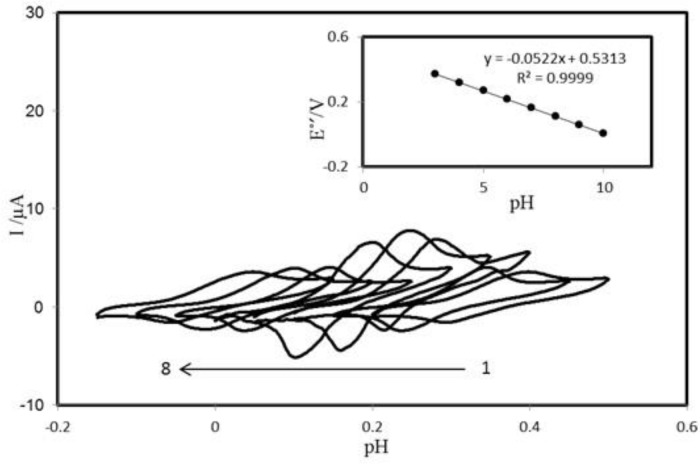
Cyclic voltammograms (at 100 mV s^−1^) of PBDCNPE at various buffered pHs. The numbers 1–8 correspond to 3, 4, 5, 6, 7, 8, 9 and 10 pHs, respectively. Inset: Plot of E°′ *vs.* pH

The voltammetric behavior of the PBDCNPE was characterized at various pHs by CV. Figure 3 shows the CVs of the modified electrode in solutions at various pH values ranging from 2.0 to 10.0. The anodic peak potential was pH dependent. The inset of Figure 3 shows Eº׳ as a function of pH. The results showed that the slope (Eº׳/pH) is -52.2 mV/pH units over a pH range from 2.0 to 10.0. This slope was close to the Nernstian value of -59 mV for a two-electron, two-proton process (40). So two protons are transferred in the redox reaction in the pH range 2.0–10.0

*Electrocatalytic oxidation of AA at a PBDCNPE*

**Figure 4:**
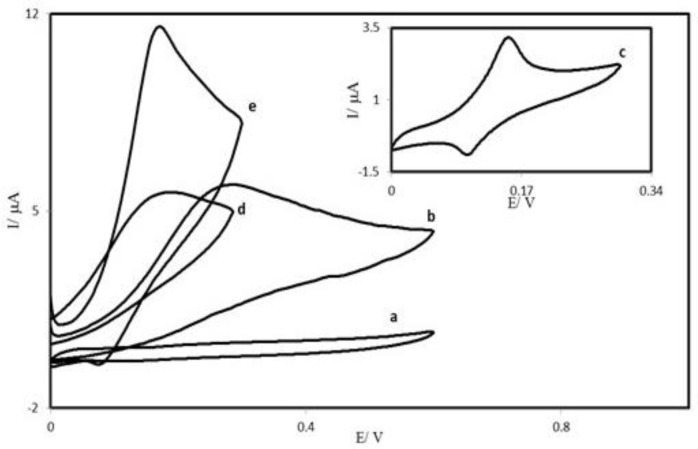
Cyclic voltammograms of: (a) an unmodified CPE in 0.1 M phosphate buffer (pH 7.0) solution and (b) the same electrode in 0.25 mM AA, pH 7.0 solution. (c) as (a) for PBDCNPE. Also, (d) and (e) as (b) at the surface of PBDCPE and PBDCNPE respectively

Figure 4 depicts the cyclic voltammetric responses from the electrochemical oxidation of 0.25 mM AA at the PBDCNPE (curve e), PBD modified CPE (PBDCPE) (curve d) and unmodified CPE (curve b). As can be seen, the anodic peak potential for AA oxidation at the PBDCNPE (curve e) and PBDCPE (curve d) was about 170 mV, while at the unmodified CPE, the peak potential was about 300 mV (curve b). From these results, it was concluded that the best electrocatalytic effect for AA oxidation was observed at the PBDCNPE (curve e). For example, the results show that the peak potential of AA oxidation at the PBDCNPE (curve e) shifted by about 130 mV toward negative values when compared with that at the unmodified carbon paste electrode (curve b).

**Figure 5:**
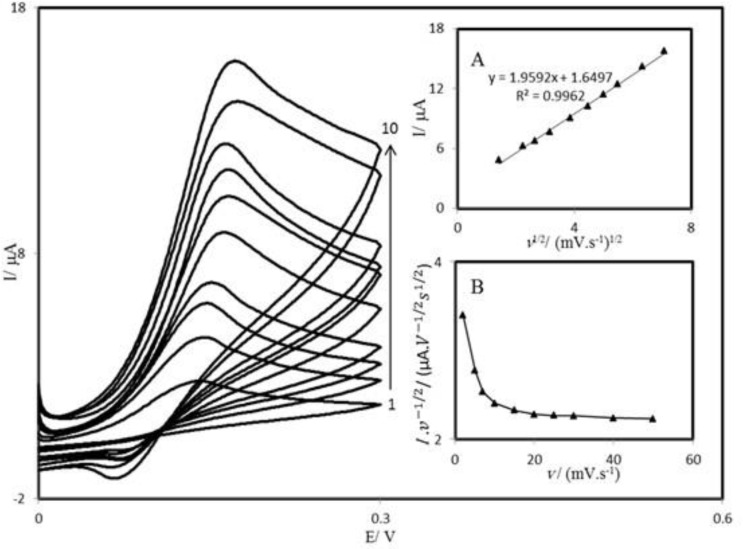
Cyclic voltammograms of a PBDCNPE in 0.1 M phosphate buffer (pH 7.00) containing 1.0 mM AA at different scan rates; the numbers 1 to 10 correspond to 2, 5, 7, 10, 15, 20, 25, 30, 40 and 50 mV s^–1^ scan rates, respectively. Insets: (A) Variation of the electrocatalytic currents *vs.* the square root of scan rate, (B) variation of the scan rate normalized current (Ip/v1/2) with scan rate.

**Figure 6:**
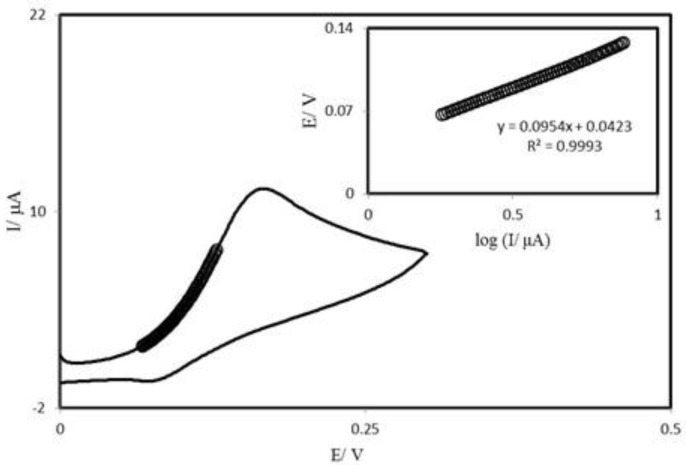
Cyclic voltammogram (at 25 mV s^−1^) of a PBDCNPE in 0.1 M phosphate buffer (pH 7.0) containing 0.25 mM AA. The points are the data used in the Tafel plot. The inset shows the Tafel plot derived from the cyclic voltammogram.

**Figure 7:**
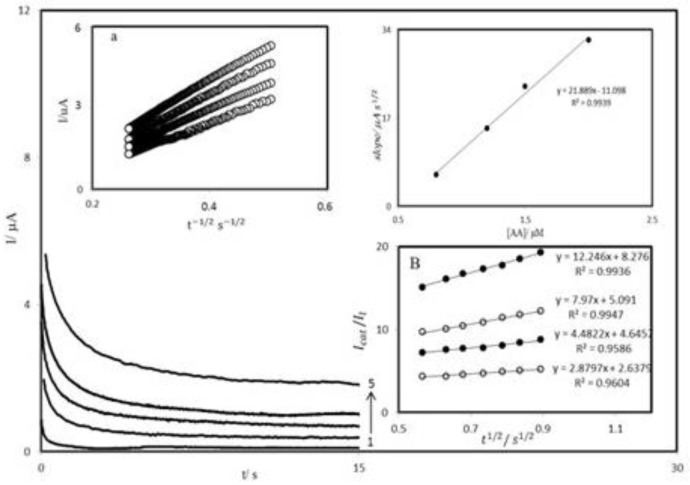
A chronoamperograms obtained at PBDCNPE in 0.1 M phosphate buffer solution (pH 7.0) for different concentration of AA. The numbers 1–5 correspond to 0.0, 0.8, 1.2, 1.4 and 2.0 mM of AA. Insets: A (a) plots of I *vs.* t^-1/2^ obtained from chronoamprograms 2–5 and A (b) plot of the slope of the straight lines against the AA concentration. B Dependence of I_cat_⁄I_l_ on t^1/2^ derived from the data of chronoamprograms shown in a.

**Figure 8:**
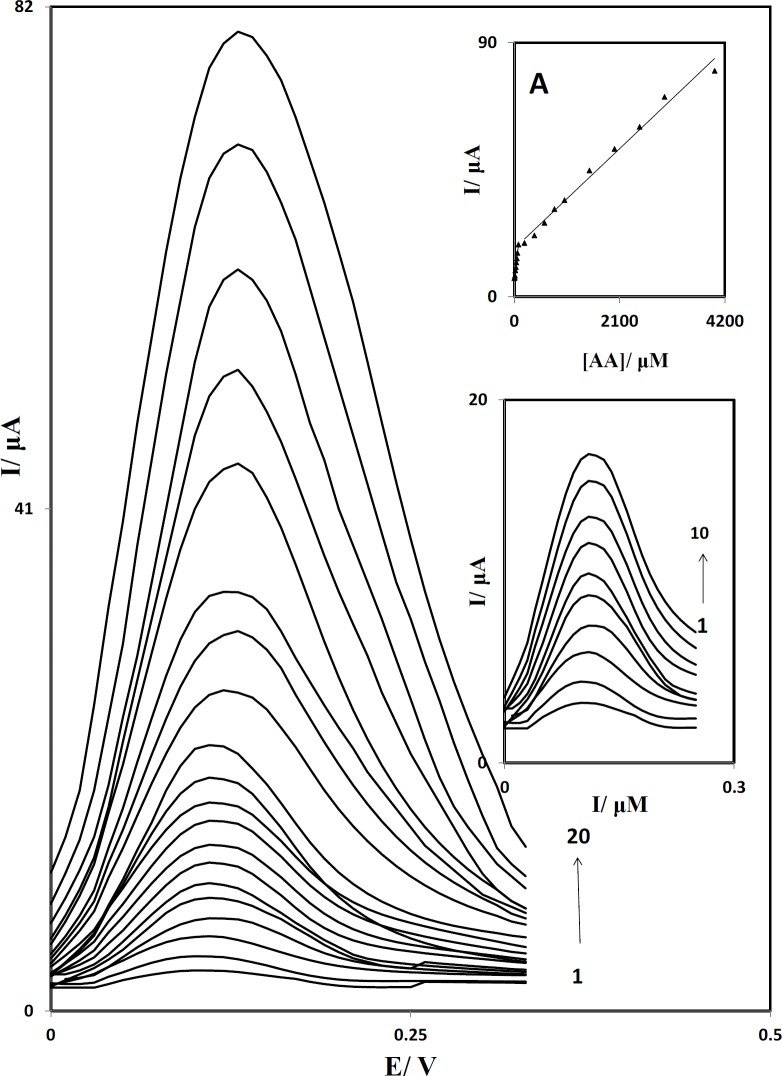
Differential pulse voltammograms of PBDCNPE in 0.1 M phosphate buffer solution (pH 7.0) containing different concentrations of AA. The numbers 1–20 correspond to: 1.0, 2.5, 5.0, 10.0, 20.0, 30.0, 40.0, 50.0, 60.0, 80.0, 200.0, 400.0, 800.0, 1000.0, 1500.0, 2000.0, 3000.0 and 4000.0 µM of AA. Inset A: The plots of the electrocatalytic peak current as a function of AA concentration. Inset B: Differential pulse voltammograms in the range of 1.0 to 80.0 µM.

**Figure 9:**
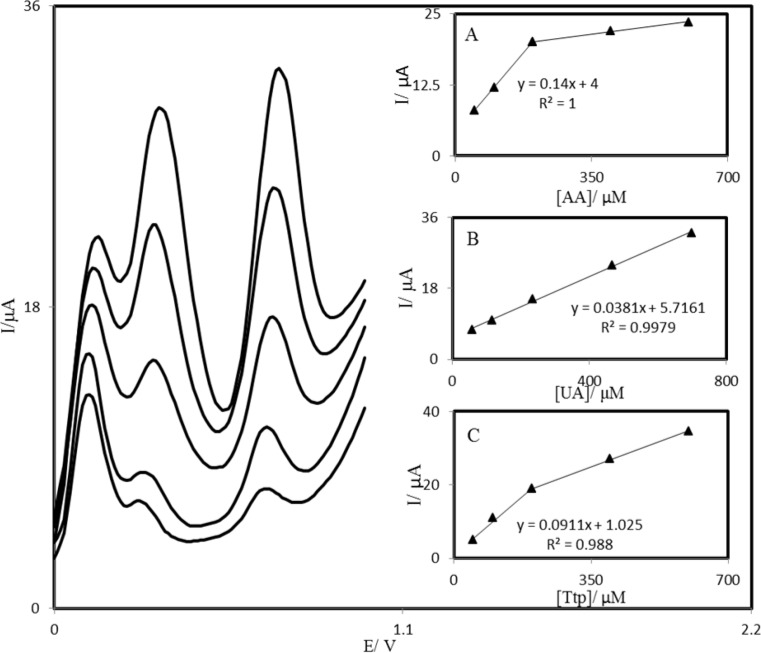
Differential pulse voltammograms of PBDCNPE in 0.1 M phosphate buffer solution (pH 7.0) containing different concentrations of AA, UA, and Trp (from inner to outer) mixed solutions of 50.0 + 58.31 + 50.0, 100.0 + 116.63 + 100.0, 200.0 + 233.3 + 200.0, 400.0 + 466.5 + 400.0 and 600.0 + 700.0 + 600.0 respectively, in which the first value is the concentration of AA in *μ*M, the second value is the concentration of UA in *μ*M, and the last value is the concentration of Trp in *μ*M. Insets: plots of the peak currents as a function of (A) AA, (B) UA, and (C) Trp concentration, respectively.

**Table 1:** Determination of Trp, UA and AA in real samples with PBDCNPE.

**Sample**	**Species**	**Added (µM)**	**Found (µM)**	**Recovery (%)**
Vitamin C tablet	AA	0	23.5	-
		10	33.1	96
		30	53.9	101.3
Urine	UA	0	9.5	-
		50	60.6	102.2
		70	78.8	99
Serum	Trp	0	ND	-
		50	51.4	102.8
		70	68.5	97.8

Similarly, when comparing the oxidation of AA at the PBDCPE (curve d) and PBDCNPE (curve e), a dramatic enhancement of the anodic peak current at the PBDCNPE relative to that obtained at the PBDCPE was observed. In other words, the data clearly show that the combination of carbon nanotube improve the characteristics of AA oxidation. The PBDCNPE, in 0.1 M phosphate buffer (pH 7.0) and without AA in solution, exhibited a well-behaved redox reaction (curve c), after addition of 0.25 mM AA, there was a dramatic enhancement of the anodic peak current (curve e), indicating a strong electrocatalytic effect (41).

*Effect of scan rate*

The scan rate dependence of cyclic voltammograms for the PBDCNPE in 0.1 M phosphate buffer solution containing 1.0 mM AA is presented in Figure 5. It can be noted from Figure 5 that, with an increasing scan rate, the peak potential for the electrooxidation of AA shifts to more positive potentials, suggesting a kinetic limitation in the reaction between the redox sites of PBD and AA.

A plot of peak height (I_p_) against the square root of scan rate (v^1/2^), in the range of 2–50 mV s^-1^, was constructed (Figure 5A), which was found to be linear, suggesting that at sufficient overpotential the process is diffusion rather than surface controlled. A plot of the sweep rate normalized current (Ip/v^1/2^) versus sweep rate (Figure 5B) exhibits the characteristic shape typical of an EC^׳^_cat_ process. 

Tafel plot was drawn from data of the rising part of the current–voltage curve recorded at a scan rate of 25 mVs^−1^ (Figure 6). This part of voltammogram, known as Tafel region, is affected by electron transfer kinetics between substrate (AA) and surface confined PBDCNPE, assuming the deprotonation of substrate as a sufficiently fast step. In this condition, the number of electron involved in the rate determining step can be estimated from the slope of Tafel plot. A slope 0.114 Vdecade^−1^ is obtained indicating a one electron transfer to be rate limiting assuming a transfer coefficient of α = 0.52.

*Chronoamperometric measurements*

The chronoamperometry was employed along with other electrochemical methods for the investigation of electrode processes at chemically modified electrodes. Figure 7A shows chronoamperometric measurements of AA at PBDCNPE. This figure represents the current–time profiles obtained by setting the working electrode potential at 350 mV for various concentrations of AA. In chronoamperometric studies, we have determined the diffusion coefficient of AA at PBDCNPE. For an electroactive material (AA in this case) with a diffusion coefficient of D, the current for the electrochemical reaction (at a mass transport limited rate) is described by the Cottrell Equation (41):

I= nFAD^1/2^C_b_π^-1/2^t^-1/2^                     (3)

Where D and C_b_ are the diffusion coefficient (cm^2 ^s^-1^) and the bulk concentration (mol cm^-3^), respectively. Under diffusion control, a plot of I versus t^-1/2^ will be linear, and from the slope the value of D can be obtained. Figure 7A inset a, shows the experimental plots with the best fits for different concentration of AA employed. The slopes of the resulting straight lines were plotted versus the AA concentration (Figure 7A inset b). The mean value of the D was found to be 4.5×10^-8 ^cm^2 ^s^-1^. Chronoamperometry can also be employed to evaluate the catalytic rate constant, k, for the reaction between AA and the PBDCNPE according to the method of Galus (42):

I_C_⁄I_L_ = γ^1/2^[ π^1/2^ erf(γ^1/2^) + exp(-γ)/ γ^1/2^]                      (4)

where I_C_ is the catalytic current of AA at the PBDCNPE, I_L_ the limited current in the absence of AA and γ = kC_b_t (C_b_ is the bulk concentration of AA) is the argument of the error function. In the cases where γ exceeds 2, the error function is almost equal to 1, and therefore, the above equation can be reduced to:

I_C_⁄I_L_ = π^1/2^γ^1/2 ^= π^1/2^(kC_b_t)^1/2^                     (5)

where t is the time elapsed (s). The above equation can be used to calculate the rate constant of the catalytic process k. Based on the slope of the I_C_⁄I_L_ versus t^1/2^ plot; k can be obtained for a given AA concentration. Such plots obtained from the chronoamperograms in Figure 7A are shown in Figure 7B. The value of k explains as well as the sharp feature of the catalytic peak observed for catalytic oxidation of AA at the surface of PBDCNPE. Finally, the heterogeneous rate constant of catalytic reaction was calculated as k = 9.3 × 10^-1^ cm s^-1^.

*Differential pulse voltammetry*

Differential pulse voltammetry (DPV) has a much higher current sensitivity and better resolution than cyclic voltammetry, there for we used Differential pulse voltammetry to determine the concentration of AA. In addition, the charging current contribution to the background current, which is a limiting factor in the analytical determination, is negligible in DPV mode. Figure 8 shows the differential pulse voltammograms obtained for the oxidation of different concentrations of AA at the PBDCNPE. The dependence of the peak current on the AA concentration is shown in inset A of Figure 8 in the range of 1.0 to 4000.0 µM. This inset clearly shows that the plot of peak current versus AA concentration is constituted of two linear segments with different slopes, corresponding to two different ranges of substrate concentration. The decrease of sensitivity (slope) in the second linear range is likely to be due to kinetic limitations. Inset B shows differential pulse voltammograms in the range of 1.0 to 80.0 µM. From the analysis of this data, we estimate that the lower limit of detection of AA is of the orde of 0.3 μM.

*Simultaneous determination of AA, UA and Trp*

The main objective of this study was to detect AA, UA, and Trp simultaneously. The utilization of the PBDCNPE for the simultaneous determination of AA, UA, and Trp was demonstrated by simultaneously changing the concentrations of AA, UA, and Trp. The AA voltammetric results showed that the simultaneous determination of AA, UA, and Trp with 3 well-distinguished anodic peaks at potentials of 100, 300, and 670 mV corresponding to the oxidation of AA, UA, and Trp, respectively, could be possible at the PBDCNPE (Figure 9). The sensitivity of the modified electrode toward the oxidation of AA was found to be 0.143 *μ*A *μ*M^−^^1^, whereas the sensitivity toward AA in the absence of UA and Trp was found to be 0.147 *μ*A *μ*M^−^^1^. It is very interesting to note that the sensitivities of the modified electrode toward AA in the absence and presence of UA and Trp were virtually the same, which indicates the fact that the oxidation processes of AA, UA, and Trp at the PBDCNPE were independent; therefore, simultaneous or independent measurements of the 3 analytes are possible without any interference. If the AA signal were affected by the UA or Trp, the above-mentioned slopes would be different.

*Interference study*

The effect of a number of organic compounds such as uric acid, folic acid, captopril, cysteine, aspartic acid, tryptophan, glysine, acetaminophen and some ions such as chloride, potassium, nitrate, fluoride, sulfide, carbonate, and sodium on the determination of 1.0 × 10^-4^ M ascorbic acid was investigated. 

The tolerance limit was taken as the maximum concentration of the foreign substances, which caused an approximately ±5% relative error in the determination. The tolerated concentration of foreign substances was 1.0 M for Na^+^, Cl^-^, F^-^, S^2-^, CO_3_^2-^, HCO_3_^-^, NO_3_^-^ and K^+^; 1.0 × 10^-3^ M for uric acid, folic acid, captopril, cysteine, aspartic acid, tryptophan, glysine and acetaminophen.

*Real sample analysis*

In order to evaluate the analytical applicability of the proposed method, it was applied to the determination of AA in vitamin C tablets, UA in urine and Trp in human serum samples. The DPV technique was used in the experiments. The differential pulse voltammograms were obtained by spiking appropriate samples in diluted solution using PBDCNPE at optimum conditions as described earlier. The results for determination of the three species in real sample are given in Table 1. From these results, it can be seen the PBDCNPE shows good activity for real samples analysis. This procedure was repeated five times and the relative standard deviation were 1.8%, 2.1% and 2.5% for AA, UA and Trp, respectively.

*The repeatability and stability of PBDCNPE*

The ability to generate a reproducible electrode surface was examined using cyclic voltammetric data from five separately prepared PBDCNPEs, obtained in optimum solution pH. The calculated RSD for various parameters (2–4%) indicated that surface reproducibility was satisfactory. This degree of reproducibility is virtually the same as that expected for an ordinary carbon paste surface (43). In addition, the long-term stability of the PBDCNPE was tested over a three-week period. When CVs were recorded after the modified electrode was stored in atmosphere at room temperature, the peak potential for AA oxidation was unchanged and the current signals showed less than 2.3% decrease relative to the initial response. The antifouling properties of the modified electrode toward AA oxidation and its oxidation products were investigated by recording the cyclic voltammograms of the modified electrode before and after use in the presence of AA. Cyclic voltammograms were recorded in the presence of AA after having cycled the potential 10 times at a scan rate of 25mV s^-1^. The peak potentials were unchanged and the currents decreased by less than 2.5%. Therefore, at the surface of PBDCNPE, not only does the sensitivity increase, but the fouling effect of the analyte and its oxidation product also decreases. The surface of the PBDCNPE was regenerated before each experiment. 

## Conclusion

A carbon paste electrode modified with PBD and oxidized carbon nanotube was fabricated and used for electrocatalytic determination of AA. The results demonstrated that the electrooxidation of AA at the surface of the PBDCNPE occurred at a potential about 130 mV less positive than that of the bare carbon paste electrode. The application of PBDCNPE for the simultaneous determination of AA, UA, and Trp was demonstrated. The detected potential differences of 200, 570, and 470 mV between AA-UA, AA-Trp, and UA-Trp, respectively, were large enough to determine AA, UA, and Trp individually and simultaneously. The proposed electrode was used in determination of AA, UA and Trp in vitamin C tablet, urine and human serum without the necessity for sample pretreatment or any time-consuming extraction or evaporation steps prior to the analysis, with satisfactory recovery. The high current sensitivity, low detection limit and high selectivity of the PBDCNPE for the detection of AA proved its potential as a sensor for practical applications.
